# The Rescue of the Romanian Health System by the Emergency Departments during the Fourth Wave of COVID-19 Pandemic

**DOI:** 10.3390/life12101547

**Published:** 2022-10-06

**Authors:** Bogdan Oprita, Andrei Davidoiu, Alexandru Bogdan Dinu, Ruxandra Oprita

**Affiliations:** 1 Faculty of Medicine, University of Medicine and Pharmacy Carol Davila Bucharest, 050474 Bucharest, Romania; 2 Emergency Department, Clinical Emergency Hospital of Bucharest, 105402 Bucharest, Romania; 3 Gastroenterology Department, Clinical Emergency Hospital of Bucharest, 105402 Bucharest, Romania

**Keywords:** COVID-19, emergency departments, fourth wave, mechanical ventilation, vaccination

## Abstract

**Simple Summary:**

The COVID-19 pandemic represents a public health problem that imposes a series of epidemiological measures with an important impact on the health system. Up to now, humanity has faced six waves of this pandemic, in each of which a certain strain of SARS-CoV-2 was dominant. In the fourth wave of the pandemic, the most presentations were recorded in emergency departments, which led to a need for special measures to allow the provision of medical care to as many patients as possible. We conducted a retrospective, observational study on a group of 1417 patients who presented themselves and received medical care at the Emergency Department of the Clinical Emergency Hospital of Bucharest, Romania, during the fourth wave of the COVID-19 pandemic. The average age of the patients included in our study (60 years) was higher than the average age reported for infection with the Omicron strains. Additionally, the severity of the cases was significant, with a rate of orotracheal intubation and mechanical ventilation of approximately 10%. In the condition of full occupancy of the hospital beds on the wards, all the necessary therapeutic measures for these patients were given in the emergency department by a multidisciplinary team.

**Abstract:**

The COVID-19 pandemic has led to the confrontation of the health system with the need to identify solutions for providing medical care to a very large number of patients. The main objective of our study was to describe the measures taken to provide optimal medical care to patients who presented themselves in one of the large emergency hospitals of Romania in the fourth wave of the COVID-19 pandemic. Material and Methods: We conducted a retrospective, observational study on a group of 1417 patients. The statistical analysis was performed using R. Results: The average length of stay of patients in the emergency departments was approximately 2.6 h, increasing to up to 15 days in some more severe cases. For rapid antigen tests, the highest positivity rate for SARS-CoV-2 was identified in patients aged >75 years (53%). Among the identified risk factors associated with the need for mechanical ventilation were advanced age (α < 0.001) and lack of vaccination against SARS-CoV-2 (α < 0.001). Discussion and conclusions: A method of saving the Romanian health system in full hospital bed occupancy conditions in the wards proved to be the provision of medical care in emergency departments.

## 1. Introduction

Up to now, humanity has faced six waves of the COVID-19 pandemic [1]. Each of these waves was characterized by a certain dominant variant of SARS-CoV-2:

- In the first wave, the ancestral strain with Asp614Gly mutation;
- In the second wave, the Beta variant (B.1.351);
- In the third wave, the Delta variant (B.1.617.2);
- In the fourth wave, the Omicron variant (B.1.1.529 and BA.1);
- In the fifth wave, the Omicron variant (BA.2);
- In the sixth wave, the Omicron variant (BA.4 and BA-5) [1,2,3].

For each of these waves, differences were reported regarding the symptoms of the patients, the severity of the disease, and the attitude of people or governments [4,5]. Anxiety about this new disease has decreased progressively, this mainly being explained by the approval of some vaccines for SARS-CoV-2 and the introduction of some alternative plans to control the pandemic [4]. However, the appearance of some mutant strains has increased the anxiety level of people again, with direct consequences on the burden of the health systems [4].

The COVID-19 pandemic imposed a series of epidemiological measures that led to consequences on the trends regarding presentation and hospitalization in medical care units. Thus, a decrease in the total number of hospitalizations was observed worldwide, but an increase in the percentage of patients that required hospitalization among those who presented themselves in emergency departments was also observed [6,7]. For example, Musseli M et al. reported for the March–May 2020 period, for Italy, a 60.4% decrease in overall visits to emergency departments, but an increase in the admission rates from 30% to 39% [6]. This phenomenon was explained by the reduced number of presentations for less severe medical conditions and the decreased ratio of non-urgent/urgent medical conditions [6]. 

Regarding hospitalization rates for patients diagnosed with COVID-19, this varies depending on the geographic region [8,9]. Broadly speaking, up to a quarter of these patients required hospital medical care, and approximately 5% required intensive therapy measures [8,9]. Under these conditions, the COVID-19 pandemic has put a lot of pressure on emergency and admission departments. According to data from the specialized literature, the negative impact of the pandemic on health care systems was felt more intensely in developing countries [10]. The higher mortality rates associated with SARS-CoV-2 infection in these regions were closely related to healthcare resources, such as the availability of staff or the number of intensive care beds [10]. Furthermore, the morbidity and mortality of patients with COVID-19 varied with the dominant SARS-CoV-2 variant in each wave but also with the immunity acquired through the disease or after vaccination [1]. 

In Romania, a developing European country, the evolution of the COVID-19 pandemic was unfavorable. During the first wave, the management of the pandemic was closely linked to the lockdown imposed by the government and the subsequent waves were more severe [11,12]. Thus, the number of patients who required hospitalization and intensive care measures increased progressively from one wave to the next [13]. In the fall of 2021, Romania faced the fourth wave of the COVID-19 pandemic [14]. At that time, the incidence of SARS-CoV-2 infection was 15 new daily cases per 100,000 inhabitants, and 20% of these patients required hospital medical care [14]. Under these conditions, the beds in the intensive care units were completely occupied quite quickly and the health system was faced with a lack of medical equipment and a lack of specialized medical assistance [15,16]. The rescue of the Romanian health system was offered at that time by the emergency departments. Thus, a significant number of patients diagnosed with COVID-19 or other medical conditions stayed and received specialized medical care in emergency departments.

The COVID-19 pandemic has put the health systems around the world in a position to find alternative measures for the care of a very large number of patients. Considering the geographical variations regarding the funding from the national state budgets for the health systems and, secondarily, the different levels of resources, the rescue measures showed variations from one region to another. The main objective of our study was to describe the measures taken to provide optimal medical care to the large number of patients who presented themselves in one of the large emergency hospitals of Romania in the fourth wave of the COVID-19 pandemic. The secondary objective was the description of some epidemiological parameters such as the positivity rate of the polymerase chain reaction (PCR) test for SARS-CoV-2, the positivity rate of SARS-CoV-2 rapid antigen test, and the vaccination rate for SARS-CoV-2 and its correlation with the severity of the case. 

## 2. Materials and Methods

### 2.1. Ethical Statement

We conducted a retrospective, observational study on a group of 1417 patients who presented themselves and received medical care in the emergency department of the Emergency Clinical Hospital of Bucharest during the fourth wave of the COVID-19 pandemic (10.08.2021–30.12.2021). The study was approved by the Ethics Committee of the Clinical Emergency Hospital of Bucharest (no 34287/24.08.2022). 

### 2.2. Data Collection

The inclusion criteria were represented by the patients who presented themselves and received medical care in the emergency department of this tertiary diagnostic and treatment center, under the conditions of full occupation of the beds on the wards and the impossibility of transfer to other hospitals for the same reasons. In conclusion, we selected the patients for whom the only medical care option could be offered by the emergency departments. A case was considered severe when endotracheal intubation was needed.

The exclusion criteria were represented by the absence of the informed consent of the patients for participation in clinical studies. None of the initially selected patients found themselves in this situation and were not excluded. 

Patient data were obtained from the patient clinical observation sheets and were centralized in an Excel database. The statistical analysis was performed using R, with several integrated packages: effects, logisticRR, ggplot2, gtsummary, and ggpubr [17,18,19,20,21,22,23]. 

### 2.3. Statistical Analysis

We used a univariate binomial logistic regression with simple and multiple variants, the dependent variable representing the absence/existence of mechanical ventilation and the independent variables represent a series of clinical-demographic parameters followed in the study. Moreover, we conducted a descriptive statistical analysis of the variables. Thus, the means and standard deviations (SD) were reported for continuous variables, considering that we have a sufficient number of patients in the sample and the central limit theorem is applicable, while absolute and relative frequencies (in percentages) were reported for categorical variables. The level of significance α of the study was 0.05; therefore, *p* values below 0.05 were considered statistically significant.

## 3. Results

### 3.1. Demographical Parameters

The gender ratio was approximately equal between men and women (754 women, 663 men) (Table 1). The average age among women was 62.7 years, with a median of 66.0 years (15.0–99.00) and the average age among men was 58.9 years, with a median of 62.0 years (0–100). The ages of the patients were divided into four categories, depending on the values of the quartiles of the variable distribution: ≤47 years old (22.81% women, 30.17% men), 48–65 years old (24.54% women, 25.64% men), 66–75 years old (25.06% women, 24.59% men) and >75 years old (27.59% women, 19.60% men) (Table 1). The average period of hospitalization of patients in emergency departments was 2.6 h, increasing up to 15 days in some more severe cases. The mortality rate among women was 1.85% and among men, it was 1.50% (Table 1). 

The vaccination against SARS-CoV-2 rate at that time turned out to be slightly higher among men than among women (32.12% among men vs. 29.58% among women) (Table 1). The highest vaccination rate was identified among patients aged between 66–75 years (40.06%), followed by the group of patients aged >75 years (34.32%), then the age category of 48–65 years (30.14%), and, finally, the age category <47 years (19.35%) (Table 2).

### 3.2. Positivity Rate for SARS-CoV-2 Infection

All patients who presented to the emergency room underwent a PCR test for SARS-CoV-2 or a rapid SARS-CoV-2 antigen test. The type of test performed depended on the epidemiological and clinical suspicion regarding the probability of establishing the diagnosis of COVID-19, and the severity of the clinical picture. Therefore, among women, 79 positive PCR tests (10.48%) and 167 positive rapid antigen tests (49.20%) were identified. Among men, these percentages were approximately similar, identifying 71 positive PCR tests (10.70%) and 318 positive rapid antigen tests (47.96%) (Table 1). In regard to age, the highest rate of PCR test positivity for SARS-CoV-2 was identified among patients aged between 48–65 years (13.52%), followed by the age category of 66–75 years (12.78%), the category of age >75 years (10.36%), and the category of age <47 years (5.91%) (Table 2). Regarding the rapid antigen test, the highest rate of positivity was identified in patients aged >75 years (52.96%), and the lowest in patients aged <47 years (43.01%) (Table 2). As far as the SARS-CoV-2 vaccination status is concerned, the positivity of the PCR tests and rapid antigen tests for SARS-CoV-2 proved to be slightly higher in vaccinated patients than non-vaccinated ones. Hence, the PCR test was positive in 13.07% of the vaccinated patients and 9.48% of the non-vaccinated patients, and the rapid antigen test was positive in 61.93% of the vaccinated patients and 42.71% of the non-vaccinated ones (Table 3).

An important element was represented by the significant percentage of patients who required mechanical ventilation: 80 women (10.61%) and 47 men (7.09%) (Table 1). We mention that we only selected patients with a positive PCR test or rapid antigen test for SARS-CoV-2 who required orotracheal intubation and received medical care in the emergency department. The rest of the patients were transferred to the intensive care unit where there were more hospital beds available for patients without SARS-CoV-2 infection. The medical team that dealt with these patients was multidisciplinary, including emergency medicine doctors, intensive care doctors, internists, pulmonologists, cardiologists, gastroenterologists, nephrologists, and surgeons.

### 3.3. Risk Factors for Orotracheal Intubation

The statistical analysis indicates that there is a strong association between the age category and the need for mechanical ventilation. Thus, compared to patients younger than or equal to 47 years old, patients in the age category of 48–65 years have odds (probability) of mechanical ventilation 6.67 times greater, patients in the age category of 66–75 years have 12.1 times greater odds of mechanical ventilation, while patients over 75 years have 19.1 times greater odds of mechanical ventilation, all these associations being statistically significant (Table 4).

In regard to gender, the statistical analysis indicates that men have odds of mechanical ventilation that are 46% lower than that of women, and the association is statistically significant (*p* = 0.021; CI 0.64 (0.44, 0.93)) (Table 5).

Another factor associated with the need for mechanical ventilation proved to be vaccination status. As such, the statistical analysis indicates that non-vaccinated patients have 2.34 times higher odds of mechanical ventilation compared to vaccinated patients, and the effect is statistically significant (*p* < 0.001) (Table 6).

Later, we performed a multiple univariate binomial logistic regression model, with all predictors for the need for mechanical ventilation. This statistical analysis excluded gender from the predictive factors for mechanical ventilation, but advanced age and a non-vaccinated status were considered risk factors (Table 7).

## 4. Discussion and Conclusions

The emergency department plays an important role in diagnostic and therapeutic management, as it is the first place where the patient comes in contact with the hospital [24]. In critical situations, when there is a significant increase in the number of patients who present themselves to the emergency room in a short time, the reorganization of emergency departments and an approach based on adaptive measures that allow the provision of medical care to as many patients as possible is required [25,26]. This kind of situation was seen during the COVID-19 pandemic which, through the epidemiological measures imposed, led to the reduction in the number of hospital beds available on the wards and the emergency departments facing a large number of patients. Particularly, the fourth wave of the COVID-19 pandemic recorded the highest number of presentations in emergency departments since the beginning of the pandemic [27]. This pandemic has strengthened the important role of emergency medicine in public health through one of its essential functions, namely the identification of patients at high risk of infection and their early isolation [24].

The emergency department of the Clinical Emergency Hospital of Bucharest was reorganized during the COVID-19 pandemic into specific areas arranged for patients infected with SARS-CoV-2 and uninfected patients. During the fourth wave of the COVID-19 pandemic, antigen tests for SARS-CoV-2 could be performed quickly for all patients who presented themselves to the emergency room. In case the clinical suspicion of COVID-19 was very high and the rapid antigen test was negative, the investigation continued with the SARS-CoV-2 PCR test. Additionally, patients who needed immediate surgical intervention or other invasive therapeutic intervention could benefit from the rapid PCR test. These measures led to an efficient triage of patients, with the isolation of those SARS-CoV-2 infected from those who did not present this infection. Another problem faced by the emergency department during this period was the large number of patients who presented themselves to the emergency room and the full occupancy of the hospital beds on the wards, associated with the impossibility of transferring these patients to other hospitals for the same reasons. Under these conditions, the solution adopted was the hospitalization of the patients and the provision of medical care in the emergency department. The severity of the cases was significant, with approximately 10% of the patients requiring mechanical ventilation. The medical care of these patients was provided by the doctors from the emergency department in collaboration with doctors from other departments. Even under these conditions, the mortality rate of these patients did not exceed 1.85%, agreeing with the data from the specialized literature. A study published in 2022 reported an increase in the in-hospital mortality rate in Romania from 2.1% in 2019 to 3.6% in 2020 [28]. However, in 2019, in-hospital deaths represented 33% of all deaths in Romania, while in 2020, they represented 31.1% [28]. 

The demographic analysis of the patients included in the study identified an approximately equal ratio between men and women. The average age among women was slightly higher than that among men (62.7 years vs. 58.9 years). Contrary to the data from the specialized literature, the average age of the patients included in our study was significantly higher [29,30]. The European Center for Disease Prevention and Control (ECDPC) reported the age of 30 years (20–33 years) as the average age of patients with Omicron infection [31]. Only 7% of the patients infected with the Omicron strain were >60 years old and 50% of them were men. Romania is a country with an aging population. Epidemiological studies claim that by 2050 the proportion of people aged >65 years in Romania will reach approximately 30% [32]. Another explanation for this result could also be found in the ECDC reports that identify Romania among the states where the Omicron strain was isolated but without dominating during the fourth wave (37.8%) [33]. The average period of hospitalization of these patients in the emergency department was 2.6 h, increasing up to 15 days in some cases.

One of the epidemiological parameters analyzed was the rate of vaccination against SARS-CoV-2 among the patients included in the study, which was approximately 30%. The differences in the vaccination rate between the sexes were relatively insignificant. With respect to age, however, the vaccination rate showed variations from 19.35% in patients aged ≤47 years to 40.06% in the 66–75 age group. Currently, the rate of vaccination against SARS-CoV-2 in Romania among the general population is approximately 42.1%, with variations between rural (29.69%) and urban areas (41.69%) [34,35]. These data indicate a slight increase in the vaccination rate in Romania from the moment our study was initiated until now. However, compared to the global vaccination rate of 62.9%, the vaccination against SARS-CoV-2 rate in Romania remains significantly lower [35].

Another analyzed parameter was the positivity rate of rapid antigen tests and PCR tests for SARS-CoV-2. Considering that only some of the patients underwent PCR tests, significant differences were identified between their positivity rate (approximately 10%) and the positivity rate of the rapid antigen test (approximately 50%). These percentages indicate the large number of patients who presented SARS-CoV-2 infection during the fourth wave of the pandemic and concur with the data from the specialized literature. Thus, Iuliano AD et al. report the fourth wave of the pandemic as the period with the highest number of presentations in emergency departments and the most hospitalizations for COVID-19 since the beginning of the pandemic [27]. However, the severity of the disease induced by the Omicron strain in wave four proved to be less compared to the severity of the disease related to the Delta strain [36,37,38]. This can also be explained by the higher rate of vaccination against SARS-CoV-2 rate in wave four [39]. Surprisingly, our study identified a higher incidence of detection of SARS-CoV-2 infection among vaccinated patients compared to non-vaccinated ones. However, we mention that in some of these patients the discovery of the SARS-CoV-2 infection was incidental, as they did not present symptoms specific to the disease. Singanayagam et al. explain this phenomenon by the fact that the vaccine reduces the risk of disease, accelerating viral clearance but not necessarily contacting the virus [40]. Another study that evaluated 565 nasopharyngeal exudates from patients infected with SARS-CoV-2 outlined the hypothesis that the infectious viral load (VL) in fully vaccinated individuals was lower in those infected with the Omicron strains compared to those infected with the Delta strain [41]. However, the number of infection cases was higher in wave four, in which the Omicron strains dominated, compared to wave three, in which the Delta strain dominated. This suggests that other mechanisms than VL growth are incriminated in the high infectivity of the Omicron strains [41].

Another important result of our study is the significant severity of the cases that received medical care in the emergency department, with approximately 10% of patients requiring orotracheal intubation and mechanical ventilation. A retrospective study carried out over 14 years in the United States highlighted an incidence of presentation in emergency departments at 371.9 per 1000 inhabitants (95% CI, 346.5–397.3) and an incidence of the need for mechanical ventilation in their rank of 0.85 per 1000 inhabitants (95%CI, 0.75–0.95) [42]. Thus, approximately 240,000 patients require mechanical ventilation annually in the USA, representing approximately 0.23% of all visits to emergency departments [42]. Regarding COVID-19, a study published in 2020 Montrief T et al. reported a rate of 10–20% regarding the need to provide specific measures to the intensive care unit, 3–10% for the need for mechanical ventilation [43]. Another study that comparatively evaluated the need for mechanical ventilation between the first four waves of the COVID-19 pandemic reported the following percentages: 16.3% in wave one, 8% in wave two, 12.4% in wave three, and 1.6% in wave four [40]. An important thing to mention is that the average age of patients from wave four included in the study was approximately 36 years [44]. In our study, the need for mechanical ventilation was higher (10%). This can be explained by the higher average age of the patients (60 years) and the increase in the number of comorbidities. Additionally, our study demonstrated an increase in the rate of mechanical ventilation with age in patients over 75 years old, this being approximately 19 times higher than patients aged ≤47 years old (α < 0.001). Another explanation is offered by the ECDC, which reports that Romania is among the countries where the Omicron strain did not dominate during the fourth wave of the COVID-19 pandemic, along with Bulgaria, Croatia, Estonia, Latvia, Poland, and Slovakia [33]. Thus, some of these cases were most likely due to other strains of SARS-CoV-2. Feikin DR et al. outline the hypothesis that in many cases, the hospitalization of patients who associate infection with the Omicron variant is due to the exacerbation of their comorbidities induced by the infection rather than COVID-19 itself [45].

Another important conclusion of our study is the increase in the rate of mechanical ventilation by 2.34 times among unvaccinated patients as compared to vaccinated ones (α < 0.001). This result validates the data from specialized literature, according to which there is an inversely proportional relationship between vaccination and the severity of Omicron infection [46,47,48,49]. The effectiveness of the vaccine for infection with the Omicron strain proved to be significantly higher in patients who had three doses of the vaccine, compared to those who had only two doses [47,49]. 

The limitations of our study are represented by the absence of data such as comorbidities and the number of vaccine doses given to each patient among the patients included in the study.

In conclusion, the emergency department offered the possibility of providing medical care to a significant number of patients in the fourth wave of the COVID-19 pandemic. The severity of these cases was variable, imposing a wide range of therapeutic measures, including orotracheal intubation. The possibility of working in multidisciplinary teams allowed the adoption of this adaptive measure, with important benefits for the patient. The volume of work in healthcare settings has increased, but it has allowed the saving of multiple human lives. All these patients received the therapeutic measures that they would have received in the hospital wards. The mortality rate among the patients included in our study did not exceed the in-hospital mortality rate reported in the specialized literature in the conditions of providing medical care in specific departments. This can be a model for reorganizing emergency departments in disaster situations when the number of patients exceeds the capacity of hospitals. We can conclude that the novelty and main impact of our study consists in the possibility of providing medical care in disaster situations for a significantly long period of time in emergency departments with good clinical results.

## Figures and Tables

**Table 1:** Demographic data of the patients included in the study related to sex.

Sex	F (*N* = 754)	M (*N* = 663)
Age categories		
≤47 years old	172 (22.81%)	200 (30.17%)
48–65 years old	185 (24.54%)	170 (25.64%)
66–75 years old	189 (25.06%)	163 (24.59%)
>75 years old	208 (27.59%)	130 (19.60%)
Age		
Average age (SD)	62.7 (18.7)	58.9 (19.1)
Median (min, max)	66.0 (15.0, 99.0)	62.0 (0, 100)
Vaccine against SARS-CoV-2		
No	531 (70.42%)	450 (67.88%)
Yes	223 (29.58%)	213 (32.12%)
PCR		
Unfulfilled	546 (72.41%)	481 (72.55%)
Inconclusive	119 (15.78%)	102 (15.38%)
Positive	79 (10.48%)	71 (10.70%)
Negative	10 (1.33%)	9 (1.37%)
Rapid Antigen Test		
Unfulfilled	216 (28.65%)	205 (30.92%)
Positive	371 (49.20%)	318 (47.96%)
Negative	167 (22.15%)	140 (21.12%)
The need for MV with OTI		
No	674 (89.39%)	616 (92.91%)
Yes	80 (10.61%)	47 (7.09%)
Death		
Yes	14 (1.86%)	10 (1.51%)
No	740 (98.14%)	653 (98.49%)

MV = mechanical ventilation; OTI = orotracheal intubation

**Table 2:** Demographic data of the patients included in the study related to age categories.

Age Categories	≤47 Years Old (*N* = 372)	48–65 Years Old (*N* = 355)	66–75 Years Old (*N* = 352)	>75 Years Old (*N* = 338)
Sex				
F	172 (46.24%)	185 (52.11%)	189 (53.69%)	208 (61.54%)
M	200 (53.76%)	170 (47.89%)	163 (46.31%)	130 (38.46%)
Vaccine against SARS-CoV-2				
No	300 (80.65%)	248 (69.86%)	211 (59.94%)	222 (65.68%)
Yes	72 (19.35%)	107 (30.14%)	141 (40.06%)	116 (34.32%)
PCR				
Unfulfilled	278 (74.73%)	251 (70.70%)	249 (70.73%)	249 (73.67%)
Inconclusive	67 (18.01%)	54 (15.21%)	53 (15.06%)	47 (13.91%)
Positive	22 (5.91%)	48 (13.52%)	45 (12.78%)	35 (10.36%)
Negative	5 (1.34%)	2 (0.56%)	5 (1.42%)	7 (2.07%)
Rapid Antigen Test				
Unfulfilled	119 (31.99%)	106 (29.86%)	99 (28.13%)	97 (28.70%)
Positive	160 (43.01%)	170 (47.89%)	180 (51.14%)	179 (52.96%)
Negative	93 (25.00%)	79 (22.25%)	73 (20.74%)	62 (18.34%)
The need for OTI with MV				
No	368 (98.92%)	331 (93.24%)	311 (88.35%)	280 (82.84%)
Yes	4 (1.08%)	24 (6.76%)	41 (11.65%)	58 (17.16%)

MV = mechanical ventilation; OTI = orotracheal intubation.

**Table 3:** Demographic data of the patients included in the study related to vaccination against SARS-CoV-2 status.

Vaccine Against SARS-CoV-2	No (*N* = 981)	Yes (*N* = 436)
Age Categories		
≤47 years old	300 (30.58%)	72 (16.51%)
48–65 years old	248 (25.28%)	107 (24.54%)
66–75 years old	211 (21.51%)	141 (32.34%)
>75 years old	222 (22.63%)	116 (26.60%)
Age		
Average (SD)	59.1 (19.7)	65.0 (16.4)
Median (min, max)	62.00 (1.00, 100)	68.50 (0, 95.0)
Sex		
F	531 (54.13%)	223 (51.15%)
M	450 (45.87%)	213 (48.85%)
PCR		
Unfulfilled	666 (67.98%)	361 (82.80%)
Inconclusive	204 (20.80%)	17 (3.90%)
Positive	93 (9.48%)	57 (13.07%)
Negative	18 (1.83%)	1 (0.23%)
Rapid Antigen Test		
Unfulfilled	282 (28.75%)	139 (31.88%)
Positive	419 (42.71%)	270 (61.93%)
Negative	280 (28.54%)	27 (6.19%)
The need for MV with OTI		
No	916 (93.37%)	371 (85.09%)
Yes	62 (6.33%)	65 (14.91%)

MV = mechanical ventilation; OTI = orotracheal intubation.

**Table 4:** The influence of age on the need for mechanical ventilation.

Predictor	*N*	OTI with MV	OR (95% CI) ^1^	*p* Value
Age categories	1417			
≤47 years old	372	4	—	
48–65 years old	355	24	6.67 (2.55–22.9)	<0.001
66–75 years old	352	41	12.1 (4.83–40.7)	<0.001
>75 years old	338	58	19.1 (7.72–63.4)	<0.001

^1^ OR = odds ratio, CI = confidence interval, MV = mechanical ventilation, OTI = orotracheal intubation.

**Table 5:** The influence of sex on the need for mechanical ventilation.

Predictor	*N*	MV with OTI	OR (95% CI) ^1^	*p* Value
Sex	1417			
F	754	80	—	
M	663	47	0.64 (0.44–0.93)	0.021

^1^ OR = odds Ratio, CI = confidence Interval, MV = mechanical ventilation, OTI = orotracheal intubation.

**Table 6:** The influence of the vaccination against SARS-CoV-2 status on the need for mechanical ventilation.

Predictor	*N*	OTI with MV	OR (95% CI) ^1^	*p* Value
Vaccination against SARS-CoV-2	1417			
Yes	436	65	—	
No	981	62	2.34 (1.61–3.38)	<0.001

^1^ OR = odds ratio, CI = confidence interval, MV = mechanical ventilation, OTI = orotracheal intubation.

**Table 7:** Multiple univariate binominal logistic regression model, with all predictors for the need for mechanical ventilation.

Predictor	OR (95% CI) ^1^	*p* Value
Age categories		
≤47 years old	—	
48–65 years old	6.07 (2.31–20.8)	<0.001
66–75 years old	10.2 (4.04–34.3)	<0.001
>75 years old	16.50 (6.64–54.90)	<0.001
Sex		
F	—	
M	0.72 (0.48–1.06)	0.10
Vaccination against SARS-CoV-2		
Yes	—	
No	1.99 (1.36–2.91)	<0.001

^1^ OR = odds ratio, CI = confidence interval.

## Data Availability

Not applicable.
